# 1-[2-(3,4-Dichloro­benz­yloxy)-2-phenyl­ethyl]-1*H*-benzimidazole

**DOI:** 10.1107/S1600536808022629

**Published:** 2008-07-26

**Authors:** Özden Özel Güven, Taner Erdoğan, Simon J. Coles, Tuncer Hökelek

**Affiliations:** a Department of Chemistry, Zonguldak Karaelmas University, 67100 Zonguldak, Turkey; b Department of Chemistry, Southampton University, Southampton SO17 1BJ, England; c Department of Physics, Hacettepe University, 06800 Beytepe, Ankara, Turkey

## Abstract

In the mol­ecule of the title compound, C_22_H_18_Cl_2_N_2_O, the planar benzimidazole ring system is oriented with respect to the phenyl and dichloro­benzene rings at dihedral angles of 12.73 (3) and 36.57 (4)°, respectively. The dihedral angle between the dichloro­benzene and phenyl rings is 29.95 (6)°. There are C—H⋯π contacts between the benzimidazole and dichloro­benzene rings, between the benzimidazole and phenyl rings, and between a methylene group and the dichlorobenzene ring.

## Related literature

For general background, see: Brammer & Feczko (1988 ▶); Özel Güven *et al.* (2007*a* ▶,*b* ▶). For related literature, see: Song & Shin (1998 ▶); Freer *et al.* (1986 ▶); Peeters *et al.* (1996 ▶, 1979*a* ▶,*b* ▶); Caira *et al.* (2004 ▶); Özel Güven *et al.* (2008*a* ▶,*b* ▶).
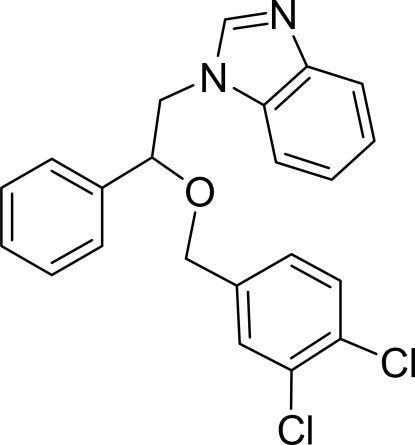


## Experimental

### 

#### Crystal data

C_22_H_18_Cl_2_N_2_O
*M* *_r_* = 397.28
Monoclinic, 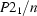

*a* = 14.4664 (3) Å
*b* = 7.3995 (2) Å
*c* = 19.1030 (3) Å
β = 111.653 (1)°
*V* = 1900.57 (7) Å^3^
*Z* = 4
Mo *K*α radiation
μ = 0.36 mm^−1^
*T* = 120 (2) K
0.40 × 0.40 × 0.30 mm

#### Data collection

Bruker–Nonius Kappa CCD diffractometer
Absorption correction: multi-scan (*SADABS*; Sheldrick, 2007 ▶) *T*_min_ = 0.871, *T*_max_ = 0.901
23210 measured reflections
4358 independent reflections
3480 reflections with *I* > 2σ(*I*)
*R*_int_ = 0.044

#### Refinement

*R*[*F* ^2^ > 2σ(*F* ^2^)] = 0.049
*wR*(*F* ^2^) = 0.137
*S* = 1.09
4358 reflections
245 parameters
H-atom parameters constrained
Δρ_max_ = 1.00 e Å^−3^
Δρ_min_ = −0.45 e Å^−3^

### 

Data collection: *COLLECT* (Hooft, 1998 ▶); cell refinement: *DENZO* (Otwinowski & Minor, 1997 ▶) and *COLLECT*; data reduction: *DENZO* and *COLLECT*; program(s) used to solve structure: *SHELXS97* (Sheldrick, 2008 ▶); program(s) used to refine structure: *SHELXL97* (Sheldrick, 2008 ▶); molecular graphics: *ORTEP-3 for Windows* (Farrugia, 1997 ▶); software used to prepare material for publication: *WinGX* (Farrugia, 1999 ▶) and *PLATON* (Spek, 2003 ▶).

## Supplementary Material

Crystal structure: contains datablocks I, global. DOI: 10.1107/S1600536808022629/dn2369sup1.cif

Structure factors: contains datablocks I. DOI: 10.1107/S1600536808022629/dn2369Isup2.hkl

Additional supplementary materials:  crystallographic information; 3D view; checkCIF report

## Figures and Tables

**Table 1:** Hydrogen-bond geometry (Å, °)

*D*—H⋯*A*	*D*—H	H⋯*A*	*D*⋯*A*	*D*—H⋯*A*
C3—H3⋯*Cg*3^i^	0.93	2.87	3.583 (2)	135
C8—H8*A*⋯*Cg*4^ii^	0.97	2.71	3.670 (2)	171
C13—H13⋯*Cg*2^iii^	0.93	2.68	3.474 (2)	144
C18—H18⋯*Cg*1	0.93	2.78	3.380 (2)	124

Symmetry codes: (i) 
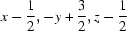
; (ii) 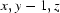; (iii) 
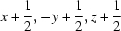
.

**Fig. 1.**
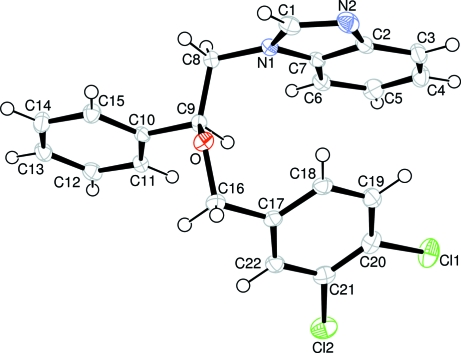
The molecular structure of the title molecule with the atom-numbering scheme. Displacement ellipsoids are drawn at the 50% probability level.

## supplementary crystallographic information

### Comment

In recent years, there has been increasing interest in synthesis of heterocyclic compounds having biological and commercial importances. Clotrimazole (Song & Shin, 1998), econazole (Freer *et al.*, 1986), ketoconazole (Peeters *et al.*, 1979*a*) and miconazole (Peeters *et al.*, 1979*b*) are well known imidazole ring containing, while itraconazole (Peeters *et al.*, 1996) and fluconazole (Caira *et al.*, 2004) are 1*H*-1,2,4-triazole ring containing, azole derivatives. They have been developed for clinical uses as antifungal agents (Brammer & Feczko, 1988). Lately, similar structures to miconazole and econazole have been reported to show antibacterial activity more than antifungal activity (Özel Güven *et al.*, 2007*a*,b). In these structures, benzimidazole ring has been found in place of the imidazole ring of miconazole and econazole. Recently, we reported the crystal structures of furyl and fluorobenzene substituted compounds (Özel Güven *et al.*, 2008*a*,b), and we report herein the crystal structure of title benzimidazole derivative.

In the molecule of the title compound (Fig. 1) the bond lengths and angles are generally within normal ranges. The planar benzimidazole ring system is oriented with respect to the phenyl and dichlorobenzene rings at dihedral angles of 12.73 (3)° and 36.57 (4)°, respectively. Atoms C8, C9 and C16 are -0.125 (2), 0.062 (2) and 0.076 (2) Å away from the ring planes of the corresponding benzimidazole, phenyl and dichlorobenzene, respectively. So, they are nearly coplanar with the adjacent rings. The dichlorobenzene ring is oriented with respect to the phenyl ring at a dihedral angle of 29.95 (6)°.

In the crystal structure, the molecules are elongated along [101], and stacked along the *b* axis. The C—H···π contacts (Table 1) between the benzimidazole and the dichlorobenzene rings, the benzimidazole and the phenyl rings and the dichlorobenzene ring and the methylene group may stabilize the structure.

### Experimental

The title compound was synthesized by the reaction of 2-(1*H*-benzimidazol-1-yl) -1-phenylethanol (Özel Güven *et al.*, 2007*a*) with NaH and appropriate benzyl halide. To the solution of alcohol (300 mg, 1.259 mmol) in DMF (2.4 ml) was added NaH (63 mg, 1.574 mmol) in small fractions. The appropriate benzyl halide (238 mg, 1.259 mmol) in DMF (1.2 ml) was added dropwise. The mixture was stirred at room temperature for 2 h, and excess hydride was decomposed with a small amount of methyl alcohol. After evaporation to dryness under reduced pressure, the crude residue was suspended with water and extracted with methylene chloride. The organic layer was dried over anhydrous sodium sulfate and then evaporated to dryness. The crude residue was purified by chromatography on a silica-gel column using chloroform-methanol as eluent. Crystals suitable for X-ray analysis were obtained by the recrystallization of the ether from a mixture of hexane/ethyl acetate (1:2) (yield; 229 mg, 46%).

### Refinement

H atoms were positioned geometrically, with C—H = 0.93, 0.98 and 0.97 Å for aromatic, methine and methylene H, respectively, and constrained to ride on their parent atoms with *U*_iso_(H) = 1.2*U*_eq_(C).

### Crystal data

**Table:**

C_22_H_18_Cl_2_N_2_O	*F*_000_ = 824
*M**_r_* = 397.28	*D*_x_ = 1.388 Mg m^−^^3^
Monoclinic, *P*2_1_/*n*	Mo *K*α radiation λ = 0.71073 Å
Hall symbol: -P 2yn	Cell parameters from 4593 reflections
*a* = 14.4664 (3) Å	θ = 2.9–27.5º
*b* = 7.3995 (2) Å	µ = 0.36 mm^−^^1^
*c* = 19.1030 (3) Å	*T* = 120 (2) K
β = 111.6530 (10)º	Block, colorless
*V* = 1900.57 (7) Å^3^	0.40 × 0.40 × 0.30 mm
*Z* = 4	

### Data collection

**Table:**

Bruker–Nonius Kappa CCD diffractometer	4358 independent reflections
Radiation source: fine-focus sealed tube	3480 reflections with *I* > 2σ(*I*)
Monochromator: graphite	*R*_int_ = 0.044
Detector resolution: 9.091 pixels mm^-1^	θ_max_ = 27.5º
*T* = 120(2) K	θ_min_ = 3.0º
φ and ω scans	*h* = −18→18
Absorption correction: multi-scan(SADABS; Sheldrick, 2007)	*k* = −9→9
*T*_min_ = 0.871, *T*_max_ = 0.901	*l* = −24→24
23210 measured reflections	

### Refinement

**Table:**

Refinement on *F*^2^	Hydrogen site location: inferred from neighbouring sites
Least-squares matrix: full	H-atom parameters constrained
*R*[*F*^2^ > 2σ(*F*^2^)] = 0.049	*w* = 1/[σ^2^(*F*_o_^2^) + (0.0784*P*)^2^ + 0.7143*P*] where *P* = (*F*_o_^2^ + 2*F*_c_^2^)/3
*wR*(*F*^2^) = 0.137	(Δ/σ)_max_ < 0.001
*S* = 1.09	Δρ_max_ = 1.00 e Å^−^^3^
4358 reflections	Δρ_min_ = −0.45 e Å^−^^3^
245 parameters	Extinction correction: SHELXL97 (Sheldrick, 2008), Fc^*^=kFc[1+0.001xFc^2^λ^3^/sin(2θ)]^-1/4^
Primary atom site location: structure-invariant direct methods	Extinction coefficient: 0.029 (2)
Secondary atom site location: difference Fourier map	

### Special details

**Table:**

Geometry. All e.s.d.'s (except the e.s.d. in the dihedral angle between two l.s. planes) are estimated using the full covariance matrix. The cell e.s.d.'s are taken into account individually in the estimation of e.s.d.'s in distances, angles and torsion angles; correlations between e.s.d.'s in cell parameters are only used when they are defined by crystal symmetry. An approximate (isotropic) treatment of cell e.s.d.'s is used for estimating e.s.d.'s involving l.s. planes.
Refinement. Refinement of *F*^2^ against ALL reflections. The weighted *R*-factor *wR* and goodness of fit *S* are based on *F*^2^, conventional *R*-factors *R* are based on *F*, with *F* set to zero for negative *F*^2^. The threshold expression of *F*^2^ > σ(*F*^2^) is used only for calculating *R*-factors(gt) *etc*. and is not relevant to the choice of reflections for refinement. *R*-factors based on *F*^2^ are statistically about twice as large as those based on *F*, and *R*- factors based on ALL data will be even larger.

### Fractional atomic coordinates and isotropic or equivalent isotropic displacement parameters (Å^2^)

**Table:**

	*x*	*y*	*z*	*U*_iso_*/*U*_eq_	
Cl1	0.08238 (4)	1.15550 (8)	0.12348 (3)	0.03377 (18)	
Cl2	0.27151 (4)	1.15173 (8)	0.27688 (3)	0.03349 (17)	
O	−0.00599 (9)	0.69157 (18)	0.39047 (7)	0.0198 (3)	
N1	−0.11880 (11)	0.5111 (2)	0.25401 (8)	0.0181 (3)	
N2	−0.25862 (12)	0.6632 (2)	0.18736 (9)	0.0248 (4)	
C1	−0.20915 (13)	0.5751 (3)	0.24963 (11)	0.0226 (4)	
H1	−0.2338	0.5577	0.2877	0.027*	
C2	−0.19558 (13)	0.6596 (2)	0.14714 (10)	0.0201 (4)	
C3	−0.20642 (15)	0.7361 (3)	0.07797 (10)	0.0256 (4)	
H3	−0.2636	0.7999	0.0502	0.031*	
C4	−0.13019 (16)	0.7146 (3)	0.05170 (10)	0.0292 (5)	
H4	−0.1367	0.7643	0.0054	0.035*	
C5	−0.04303 (16)	0.6198 (3)	0.09293 (11)	0.0268 (4)	
H5	0.0069	0.6079	0.0735	0.032*	
C6	−0.03008 (14)	0.5433 (3)	0.16255 (10)	0.0207 (4)	
H6	0.0276	0.4807	0.1904	0.025*	
C7	−0.10738 (13)	0.5649 (2)	0.18831 (9)	0.0177 (4)	
C8	−0.04415 (13)	0.4222 (3)	0.31792 (9)	0.0189 (4)	
H8A	−0.0160	0.3212	0.3002	0.023*	
H8B	−0.0753	0.3751	0.3512	0.023*	
C9	0.03905 (13)	0.5531 (2)	0.36165 (9)	0.0167 (4)	
H9	0.0662	0.6079	0.3266	0.020*	
C10	0.12201 (13)	0.4534 (2)	0.42245 (9)	0.0165 (4)	
C11	0.21552 (13)	0.4418 (3)	0.41786 (10)	0.0194 (4)	
H11	0.2275	0.4995	0.3788	0.023*	
C12	0.29134 (14)	0.3446 (3)	0.47127 (11)	0.0231 (4)	
H12	0.3537	0.3378	0.4678	0.028*	
C13	0.27398 (14)	0.2579 (3)	0.52970 (10)	0.0237 (4)	
H13	0.3246	0.1924	0.5653	0.028*	
C14	0.18091 (15)	0.2691 (3)	0.53482 (10)	0.0246 (4)	
H14	0.1692	0.2119	0.5741	0.029*	
C15	0.10499 (14)	0.3660 (3)	0.48123 (10)	0.0207 (4)	
H15	0.0426	0.3724	0.4846	0.025*	
C16	0.04829 (15)	0.8583 (3)	0.40680 (10)	0.0225 (4)	
H16A	0.1144	0.8364	0.4438	0.027*	
H16B	0.0150	0.9426	0.4286	0.027*	
C17	0.05701 (13)	0.9422 (2)	0.33712 (10)	0.0193 (4)	
C18	−0.02613 (14)	0.9540 (2)	0.27082 (10)	0.0202 (4)	
H18	−0.0877	0.9163	0.2702	0.024*	
C19	−0.01811 (14)	1.0213 (3)	0.20569 (10)	0.0221 (4)	
H19	−0.0739	1.0270	0.1614	0.027*	
C20	0.07358 (15)	1.0803 (3)	0.20677 (10)	0.0237 (4)	
C21	0.15655 (14)	1.0755 (3)	0.27335 (11)	0.0229 (4)	
C22	0.14856 (14)	1.0041 (3)	0.33816 (10)	0.0216 (4)	
H22	0.2044	0.9977	0.3824	0.026*	

### Atomic displacement parameters (Å^2^)

**Table:**

	*U*^11^	*U*^22^	*U*^33^	*U*^12^	*U*^13^	*U*^23^
Cl1	0.0443 (3)	0.0369 (3)	0.0292 (3)	0.0115 (2)	0.0242 (2)	0.0078 (2)
Cl2	0.0271 (3)	0.0344 (3)	0.0450 (3)	−0.0047 (2)	0.0204 (2)	−0.0018 (2)
O	0.0211 (6)	0.0179 (7)	0.0222 (6)	−0.0002 (5)	0.0103 (5)	0.0002 (5)
N1	0.0156 (7)	0.0187 (8)	0.0181 (7)	−0.0008 (6)	0.0042 (6)	0.0008 (6)
N2	0.0180 (8)	0.0252 (9)	0.0280 (8)	0.0022 (6)	0.0050 (7)	−0.0017 (7)
C1	0.0163 (9)	0.0259 (10)	0.0250 (9)	−0.0021 (7)	0.0067 (7)	−0.0020 (8)
C2	0.0170 (9)	0.0175 (9)	0.0208 (9)	0.0003 (7)	0.0012 (7)	−0.0028 (7)
C3	0.0264 (10)	0.0206 (10)	0.0209 (9)	0.0028 (8)	−0.0017 (7)	0.0006 (7)
C4	0.0390 (12)	0.0280 (11)	0.0175 (9)	−0.0024 (9)	0.0069 (8)	0.0020 (8)
C5	0.0321 (11)	0.0271 (11)	0.0241 (10)	−0.0015 (8)	0.0138 (8)	−0.0030 (8)
C6	0.0188 (9)	0.0189 (9)	0.0220 (9)	0.0016 (7)	0.0048 (7)	−0.0032 (7)
C7	0.0170 (8)	0.0163 (9)	0.0161 (8)	−0.0018 (7)	0.0018 (6)	−0.0018 (6)
C8	0.0191 (9)	0.0180 (9)	0.0165 (8)	−0.0013 (7)	0.0030 (7)	0.0022 (7)
C9	0.0188 (8)	0.0160 (9)	0.0151 (8)	0.0017 (7)	0.0062 (6)	0.0007 (6)
C10	0.0174 (8)	0.0161 (9)	0.0138 (8)	−0.0010 (7)	0.0031 (6)	−0.0017 (6)
C11	0.0194 (9)	0.0211 (10)	0.0160 (8)	−0.0012 (7)	0.0045 (7)	0.0005 (7)
C12	0.0182 (9)	0.0247 (10)	0.0241 (9)	0.0010 (7)	0.0050 (7)	−0.0018 (7)
C13	0.0240 (9)	0.0201 (10)	0.0207 (9)	0.0026 (8)	0.0009 (7)	0.0004 (7)
C14	0.0305 (10)	0.0237 (10)	0.0190 (9)	0.0008 (8)	0.0084 (7)	0.0045 (7)
C15	0.0201 (9)	0.0221 (10)	0.0208 (9)	0.0007 (7)	0.0086 (7)	0.0017 (7)
C16	0.0277 (10)	0.0180 (10)	0.0204 (9)	−0.0024 (7)	0.0071 (7)	−0.0016 (7)
C17	0.0223 (9)	0.0121 (9)	0.0212 (9)	−0.0007 (7)	0.0055 (7)	−0.0018 (7)
C18	0.0200 (9)	0.0151 (9)	0.0246 (9)	−0.0006 (7)	0.0073 (7)	−0.0025 (7)
C19	0.0234 (9)	0.0183 (9)	0.0221 (9)	0.0041 (7)	0.0055 (7)	−0.0012 (7)
C20	0.0337 (11)	0.0185 (10)	0.0245 (9)	0.0062 (8)	0.0172 (8)	0.0002 (7)
C21	0.0221 (9)	0.0183 (10)	0.0310 (10)	0.0010 (7)	0.0128 (8)	−0.0031 (8)
C22	0.0219 (9)	0.0167 (9)	0.0232 (9)	0.0005 (7)	0.0046 (7)	−0.0023 (7)

### Geometric parameters (Å, °)

**Table:**

Cl1—C20	1.7340 (19)	C9—H9	0.9800
Cl2—C21	1.7339 (19)	C11—C10	1.390 (2)
O—C9	1.429 (2)	C11—C12	1.391 (3)
O—C16	1.434 (2)	C11—H11	0.9300
N1—C1	1.363 (2)	C12—C13	1.388 (3)
N1—C7	1.383 (2)	C12—H12	0.9300
N1—C8	1.455 (2)	C13—H13	0.9300
N2—C1	1.313 (3)	C14—C13	1.388 (3)
N2—C2	1.393 (2)	C14—H14	0.9300
C1—H1	0.9300	C15—C10	1.394 (2)
C2—C3	1.392 (3)	C15—C14	1.392 (3)
C2—C7	1.413 (2)	C15—H15	0.9300
C3—C4	1.379 (3)	C16—H16A	0.9700
C3—H3	0.9300	C16—H16B	0.9700
C4—H4	0.9300	C17—C16	1.516 (2)
C5—C4	1.403 (3)	C18—C17	1.391 (2)
C5—H5	0.9300	C18—H18	0.9300
C6—C5	1.393 (3)	C19—C18	1.384 (3)
C6—H6	0.9300	C19—C20	1.389 (3)
C7—C6	1.387 (3)	C19—H19	0.9300
C8—H8A	0.9700	C21—C20	1.390 (3)
C8—H8B	0.9700	C22—C17	1.395 (3)
C9—C8	1.531 (2)	C22—C21	1.389 (3)
C9—C10	1.518 (2)	C22—H22	0.9300
			
C9—O—C16	114.20 (14)	C15—C10—C9	121.13 (15)
C1—N1—C7	106.09 (15)	C10—C11—C12	120.52 (17)
C1—N1—C8	127.33 (15)	C10—C11—H11	119.7
C7—N1—C8	126.25 (15)	C12—C11—H11	119.7
C1—N2—C2	103.97 (15)	C13—C12—C11	120.07 (18)
N2—C1—N1	114.69 (17)	C13—C12—H12	120.0
N2—C1—H1	122.7	C11—C12—H12	120.0
N1—C1—H1	122.7	C14—C13—C12	119.79 (17)
C3—C2—N2	130.43 (17)	C14—C13—H13	120.1
C3—C2—C7	119.48 (18)	C12—C13—H13	120.1
N2—C2—C7	110.06 (16)	C13—C14—C15	120.10 (17)
C4—C3—C2	118.26 (18)	C13—C14—H14	119.9
C4—C3—H3	120.9	C15—C14—H14	119.9
C2—C3—H3	120.9	C14—C15—C10	120.35 (17)
C3—C4—C5	121.79 (18)	C14—C15—H15	119.8
C3—C4—H4	119.1	C10—C15—H15	119.8
C5—C4—H4	119.1	O—C16—C17	112.19 (14)
C6—C5—C4	121.03 (19)	O—C16—H16A	109.2
C6—C5—H5	119.5	C17—C16—H16A	109.2
C4—C5—H5	119.5	O—C16—H16B	109.2
C7—C6—C5	116.73 (17)	C17—C16—H16B	109.2
C7—C6—H6	121.6	H16A—C16—H16B	107.9
C5—C6—H6	121.6	C18—C17—C22	119.34 (17)
N1—C7—C6	132.09 (16)	C18—C17—C16	120.09 (16)
N1—C7—C2	105.19 (15)	C22—C17—C16	120.56 (16)
C6—C7—C2	122.71 (17)	C19—C18—C17	120.66 (17)
N1—C8—C9	111.26 (15)	C19—C18—H18	119.7
N1—C8—H8A	109.4	C17—C18—H18	119.7
C9—C8—H8A	109.4	C18—C19—C20	119.73 (17)
N1—C8—H8B	109.4	C18—C19—H19	120.1
C9—C8—H8B	109.4	C20—C19—H19	120.1
H8A—C8—H8B	108.0	C19—C20—C21	120.17 (17)
O—C9—C10	113.44 (13)	C19—C20—Cl1	118.70 (15)
O—C9—C8	106.58 (14)	C21—C20—Cl1	121.12 (15)
C10—C9—C8	110.51 (15)	C22—C21—C20	119.83 (17)
O—C9—H9	108.7	C22—C21—Cl2	118.88 (14)
C10—C9—H9	108.7	C20—C21—Cl2	121.27 (15)
C8—C9—H9	108.7	C21—C22—C17	120.19 (17)
C11—C10—C15	119.17 (16)	C21—C22—H22	119.9
C11—C10—C9	119.64 (15)	C17—C22—H22	119.9
			
C16—O—C9—C10	81.33 (18)	O—C9—C10—C11	−125.62 (17)
C16—O—C9—C8	−156.82 (14)	C8—C9—C10—C11	114.75 (18)
C9—O—C16—C17	61.95 (19)	O—C9—C10—C15	57.3 (2)
C2—N2—C1—N1	−0.7 (2)	C8—C9—C10—C15	−62.3 (2)
C1—N2—C2—C3	−177.7 (2)	C10—C11—C12—C13	0.1 (3)
C1—N2—C2—C7	0.3 (2)	C12—C11—C10—C15	−0.1 (3)
C7—N1—C1—N2	0.9 (2)	C12—C11—C10—C9	−177.28 (17)
C8—N1—C1—N2	174.54 (17)	C11—C12—C13—C14	−0.3 (3)
C1—N1—C7—C6	178.1 (2)	C15—C14—C13—C12	0.5 (3)
C8—N1—C7—C6	4.3 (3)	C14—C15—C10—C11	0.3 (3)
C1—N1—C7—C2	−0.58 (19)	C14—C15—C10—C9	177.43 (17)
C8—N1—C7—C2	−174.36 (16)	C10—C15—C14—C13	−0.5 (3)
C1—N1—C8—C9	−100.7 (2)	C18—C17—C16—O	46.3 (2)
C7—N1—C8—C9	71.8 (2)	C22—C17—C16—O	−132.13 (18)
N2—C2—C3—C4	178.49 (19)	C19—C18—C17—C22	2.0 (3)
C7—C2—C3—C4	0.6 (3)	C19—C18—C17—C16	−176.48 (17)
C3—C2—C7—N1	178.44 (16)	C20—C19—C18—C17	−1.0 (3)
N2—C2—C7—N1	0.2 (2)	C18—C19—C20—C21	−1.4 (3)
C3—C2—C7—C6	−0.4 (3)	C18—C19—C20—Cl1	177.45 (14)
N2—C2—C7—C6	−178.63 (17)	C22—C21—C20—C19	2.8 (3)
C2—C3—C4—C5	−0.5 (3)	Cl2—C21—C20—C19	−178.87 (15)
C6—C5—C4—C3	0.0 (3)	C22—C21—C20—Cl1	−176.03 (15)
C7—C6—C5—C4	0.3 (3)	Cl2—C21—C20—Cl1	2.3 (2)
N1—C7—C6—C5	−178.58 (19)	C21—C22—C17—C18	−0.5 (3)
C2—C7—C6—C5	−0.1 (3)	C21—C22—C17—C16	177.89 (17)
O—C9—C8—N1	62.89 (17)	C17—C22—C21—C20	−1.8 (3)
C10—C9—C8—N1	−173.43 (14)	C17—C22—C21—Cl2	179.81 (14)

### Hydrogen-bond geometry (Å, °)

**Table:**

*D*—H···*A*	*D*—H	H···*A*	*D*···*A*	*D*—H···*A*
C3—H3···Cg3^i^	0.93	2.87	3.583 (2)	135
C8—H8A···Cg4^ii^	0.97	2.71	3.670 (2)	171
C13—H13···Cg2^iii^	0.93	2.68	3.474 (2)	144
C18—H18···Cg1	0.93	2.78	3.380 (2)	124

Symmetry codes: (i) *x*−1/2, −*y*+3/2, *z*−1/2; (ii) *x*, *y*−1, *z*; (iii) *x*+1/2, −*y*+1/2, *z*+1/2.
